# HPV16 and HPV18 seropositivity and DNA detection among men who have sex with men: a cross-sectional study conducted in a sexual health clinic in London

**DOI:** 10.1136/sextrans-2020-054726

**Published:** 2020-12-23

**Authors:** Eleanor M King, David Mesher, Pam Sonnenberg, Ezra Linley, Kavita Panwar, Simon Beddows, Kate Soldan, Ray Borrow, Mark Jit, Richard Gilson

**Affiliations:** 1 Institute for Global Health, University College London, London, UK; 2 Blood Safety, Hepatitis, Sexually Transmitted Infections (STI) and HIV Service, Public Health England, London, UK; 3 Vaccine Evaluation Unit, Public Health England, Manchester, UK; 4 Virus Reference Department, Public Health England, London, UK; 5 Modelling and Economics Unit, Public Health England, London, UK; 6 Department of Epidemiology and Population Health, London School of Hygiene & Tropical Medicine, London, UK; 7 The Mortimer Market Centre, Central and North West London NHS Foundation Trust, London, UK

**Keywords:** HPV, serology, vaccination, men, sexual health

## Abstract

**Objectives:**

Men who have sex with men (MSM) have an increased risk of human papillomavirus (HPV) infection and related diseases compared with men who have sex exclusively with women. From April 2018, there has been a phased roll-out of HPV vaccination offered to MSM aged up to 45 years old who are attending sexual health clinics and HIV clinics in England. The vaccine is most effective if delivered prior to HPV infection. We estimated the proportion of MSM with no current vaccine-type infection and no serological evidence of prior infection, in a study undertaken prior to vaccine introduction.

**Methods:**

We conducted a cross-sectional study among 484 MSM aged 18–40 years old who attended a sexual health clinic in London between 2010 and 2012. We estimated the prevalence of current and past infection by testing for HPV DNA in anogenital samples and for serum antibodies to HPV16 and HPV18.

**Results:**

The median age was 30 years (IQR 25–35). The prevalence of HPV16 and HPV18 DNA was 13.2% and 6.2%, respectively. Seropositivity for HPV16 and HPV18 was 28.5% and 17.1%, respectively, with 11.4% seropositive for both types. Seropositivity for the same HPV type was strongly associated with anogenital DNA detection. 279 MSM (57.6%) tested negative for both HPV16 and HPV18 serology and were DNA negative for these two types; only 5 MSM (1.0%) were seropositive and DNA positive for both HPV types.

**Conclusions:**

This is the first study to determine both the prevalence of HPV DNA in anogenital samples and HPV seroprevalence among MSM attending a sexual health clinic in the UK. Over half of MSM in this study had no evidence of a previous or current infection with either of the high-risk HPV types included in the quadrivalent vaccine, which supports the rationale for opportunistic HPV vaccination of MSM attending sexual health clinics.

## Introduction

In the UK, the National Human Papillomavirus (HPV) Immunisation Programme was introduced in 2008 for girls aged 12–13 years, with a catch-up programme up to 18 years old. Initially the bivalent vaccine was used, which protects against two of the high-risk types, HPV16 and HPV18, associated with cancers of the cervix, vulva, vagina, anus, penis and oropharynx.^1^ Since 2012, the national programme has used the quadrivalent vaccine, which protects against HPV16 and HPV18, as well as the low-risk types HPV6 and HPV11, which are responsible for the vast majority of genital warts.^2^ The vaccines are prophylactic and hence are most effective if given prior to exposure to HPV infection. The presence of a current HPV infection is evidenced by detectable HPV DNA. Not all individuals who have a natural infection will seroconvert for that HPV type. However, where there is serological evidence of HPV infection, this could indicate either a current infection or past infection. Both HPV DNA infection and HPV seropositivity for vaccine types can reduce the efficacy of vaccination.^3^ Therefore, it is important to understand the HPV DNA prevalence and HPV seroprevalence of a population prior to recommending a vaccination strategy. Serological studies directly contributed to the cost-effectiveness modelling that informed the introduction of female HPV vaccination in the UK.^4^ The high coverage achieved in the female vaccination programme in the UK has been shown to have a substantial impact on the prevalence of vaccine-type HPV infection in women in England^5^ and Scotland.^6^ Furthermore, data from England, Australia and elsewhere suggest a herd protection effect, with a decline in the incidence of genital warts among young women and heterosexual men.^7–10^ However, men who have sex with men (MSM) are likely to have little or no herd protection from vaccinating only women and will continue to have a high risk of HPV infection and related diseases.^11 12^

We have previously published data on the prevalence of current HPV infection, determined by HPV DNA detection, among MSM attending a London sexual health clinic.^11^ These data informed the mathematical models used to estimate the impact and cost-effectiveness of HPV vaccination of MSM in England.^13^ Subsequently, in November 2015, the Joint Committee on Vaccination and Immunisation advised that a targeted programme of HPV vaccination for MSM aged up to 45 years old attending specialist sexual health services or HIV clinics in the UK should be introduced, if it could be delivered cost-effectively.^14^ Following this advice, a pilot programme was implemented in 42 clinics across England, with the first clinic starting vaccination in June 2016. Following the success of the pilot, it was announced that there would be a phased national roll-out of the programme from April 2018. The impact of vaccination of MSM on rates of HPV infection and related diseases will depend on the proportion of eligible MSM with a current or previous HPV infection. We present here HPV16 and HPV18 seroprevalence in the same population of MSM to better estimate the potential benefit of vaccination in this setting.

## Methods

We conducted a cross-sectional survey including men aged 18–40 years old who reported anal or oral sex with another man in the last 5 years and who attended the Mortimer Market Centre in London between October 2010 and July 2012. Participants were asked to complete a computer-assisted self-interview questionnaire which included demographics, sexual behaviour, history of STIs, knowledge of HPV and attitudes towards HPV vaccination. At the same visit, specimens were collected for HPV DNA testing. Full details of sampling and HPV DNA testing have been previously published.^11^ In brief, two swabs were taken, one anal and one from the glans of the penis/coronal sulcus, penile shaft, scrotum and perianal area (external genital). Specimens were tested for type-specific HPV DNA using an inhouse multiplex PCR and Luminex-based genotyping test to detect 20 HPV types (high-risk types: 16, 18, 31, 33, 35, 39, 45, 51, 52, 56, 58, 59, 68; possible high-risk types: 26, 53, 70, 73, 82; and low-risk types: 6, 11).

A blood sample was also taken at the same time as the genital specimens and used to produce two aliquots of serum stored at −20°C before transportation on dry ice to Public Health England Vaccine Evaluation Unit, Manchester. Serum specimens were tested for IgG to HPV16 and HPV18 using a type-specific ELISA as described previously.^15^ Results are expressed as ELISA units per millilitre (EU/mL) by reference to a standard, with cut-offs for detection of 19 and 18 EU/mL for HPV16 and HPV18, respectively.

The association between type-specific HPV DNA detection (at any anogenital site) and HPV seropositivity was explored using logistic regression. This analysis was performed separately for HPV16 and HPV18. Regression analyses were adjusted for demographic and sexual behaviour variables which potentially confounded the association between HPV DNA detection and HPV seropositivity (the final model for both HPV types was adjusted for by age and lifetime number of male sexual partners, categorised as <20 men, 21–30 men, 31–100 men, 101–500 men and more than 500 men).

## Results

A total of 522 MSM participated in the study. These analyses are restricted to the 484 (92.7%) MSM with at least one adequate anal/external genital swab and a blood sample. The median age was 30 years (IQR 25–35). Demographic and behavioural characteristics of study participants are shown in table 1. The prevalence of high-risk HPV DNA detection in anogenital swabs was 47.3% (n=229; 95% CI 42.9% to 51.8%). HPV16 and HPV18 prevalence was 13.2% (95% CI 10.5% to 16.6%) and 6.2% (95% CI 4.4% to 8.7%), respectively; 1.5% (95% CI 0.7% to 3.0%) of MSM were HPV DNA positive for both HPV16 and HPV18. Full details of the HPV DNA prevalence data have been previously published.^11^

**Table 1:** Patient characteristics and sexual behaviour among MSM with valid blood sample and at least one eligible anal/external genital sample

	n (%)
Age group (years)	
18–20	16 (3.3)
21–25	114 (23.6)
26–30	122 (25.2)
31–35	134 (27.7)
36–40	98 (20.3)
Ethnicity	
White	358 (76.7)
Black	28 (6)
Asian and South-East Asian	34 (7.3)
Mixed/other	47 (10.1)
Country of birth	
UK	220 (46.8)
Outside of UK	250 (53.2)
Smoker	
No	330 (70.4)
Yes	139 (29.6)
Alcohol use†	
No-risk drinking	162 (34.8)
Risk drinking	303 (65.2)
Currently employed	
No	102 (21.7)
Yes	368 (78.3)
Years of education since 16 years of age	
None	12 (2.6)
Up to 2 years	57 (12.1)
3 years or more	319 (67.9)
Currently studying	82 (17.5)
Sexual orientation	
Straight/heterosexual	0 (0)
Gay/homosexual	427 (91)
Bisexual	42 (9)
Total lifetime male sex partners*	
≤30	161 (34.3)
31–100	161 (34.3)
101–500	148 (31.5)
HIV-positive	
No	459 (94.8)
Yes	25 (5.2)

*Includes oral and anal partners.

†Alcohol use disorders identification test (AUDIT-C)

MSM, men who have sex with men.

**Figure 1:**
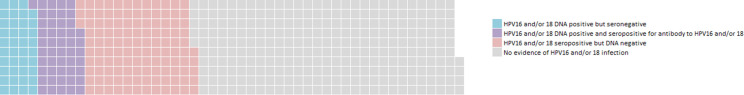
HPV DNA infection and HPV seropositivity among 484 men who have sex with men. HPV, human papillomavirus.

The concordance between HPV DNA detection and type-specific seropositivity is shown in table 2 and figure 1. Of the 484 participants, 28.5% (n=138) were seropositive for HPV16, 17.1% (n=83) for HPV18 and 11.4% (n=55) seropositive for both HPV types. Around two-thirds of MSM (n=318; 65.7%) were seronegative for both HPV16 and HPV18.

**Table 2:** Concordance between HPV DNA detection and HPV seroprevalence for HPV16 and HPV18 among eligible MSM

Seroprevalence	HPV DNA detection			
	HPV16-negative/HPV18-negative	HPV16-positive/HPV18-negative	HPV16-negative/HPV18-positive	HPV16-positive/HPV18-positive
Seronegative for both HPV16 and HPV18	279 (57.6)	25 (5.2)	13 (2.7)	1 (0.2)
HPV16 seropositive and HPV18 seronegative	58 (12.0)	21 (4.3)	4 (0.8)	0 (0.0)
HPV16 seronegative and HPV18 seropositive	24 (5.0)	0 (0.0)	3 (0.6)	1 (0.2)
Seropositive for both HPV16 and HPV18	36 (7.4)	11 (2.3)	3 (0.6)	5 (1.0)
Total	397	57	23	7

Number in brackets represents percentage of the total eligible samples (N=484).

Grey shaded cells are the numbers with fully concordant serology and HPV DNA detection results.

HPV, human papillomavirus; MSM, men who have sex with men.

Of the 138 MSM who were seropositive for HPV16, 37 (26.8%) had concurrent HPV16 DNA detected and only 12 (8.7%) had HPV18 DNA detected (table 2). Seropositivity for HPV16 was associated with anogenital HPV16 DNA detection (OR 4.33; 95% CI 2.51 to 7.46). Similarly, of 83 MSM who were seropositive for HPV18, 12 (14.4%) had concurrent HPV18 DNA detected while 17 (20.5%) had HPV16 DNA detected (table 2). Seropositivity for HPV18 was also associated with anogenital HPV18 DNA detection (OR 3.60; 95% CI 1.66 to 7.79). Only five MSM (1.0%) were both seropositive for both HPV16 and HPV18 and DNA positive for both viruses.

No evidence of either current or past HPV16 or HPV18 infection was found in 57.6% of MSM (n=279).

## Discussion

We present the results of tests for serum HPV antibody against two high-risk HPV types in a population of MSM attending a London sexual health clinic who were also tested for anogenital HPV DNA. Compared with data on HPV DNA prevalence alone, this provides a more precise estimate of the potential benefit of vaccinating this population. Over half of the MSM had no evidence of either a previous or current HPV16 or HPV18 infection, based on the results of both HPV DNA and serology. Around one-third (35.0%) had antibody to either or both HPV types, and a further 8.2% had detectable HPV DNA but negative serology.

This is the first study to relate the prevalence of HPV DNA and HPV type-specific antibody among MSM in England, and importantly was conducted among sexual health clinic attenders who would now be eligible to receive the HPV vaccine. A major strength of the study was that we compared HPV DNA prevalence at multiple genital sites with HPV seroprevalence using samples collected at the same time. We found that the majority of seropositive MSM were DNA negative for the same HPV type, suggesting that they either had an infection which was undetected at the time or they had a previous HPV infection that was cleared.

Not all men will seroconvert following a natural infection with an HPV type, although studies of HPV seroprevalence among MSM^16–18^ have demonstrated that this is higher than seroprevalence among men who have sex with women.^17 19 20^ This is likely in part to be due to higher risk sexual behaviour and in part due to the site of infection, with evidence that infection of the mucosal epithelium of the anal canal may be more likely to induce an antibody response, compared with infection of the external genitalia, which usually involves keratinised squamous epithelium. This is supported by the observation that HPV seropositivity was associated with anal HPV infection, but not external genital infection.^18 21 22^

HPV vaccines are licensed for prophylaxis, but not for therapy of existing infections, and it has been shown that men with no current HPV infection but who are seropositive for an HPV type at the time of vaccination have a reduced vaccine efficacy against that specific type compared with those who are seronegative.^3^ However, MSM with evidence of present or past infection with some, but not all, vaccine-type HPV infections still could benefit from vaccination to protect against those types that they have not already been infected with, particularly if their risk of HPV infection remains high. It is not clear whether vaccination of those with a prior infection has an impact on reinfection with the same HPV type, although there is evidence to suggest that the immune response to natural infection differs in both specificity and B cell memory from that induced by vaccination.^23 24^ A study in patients with genital warts, caused by low-risk HPV types, has provided some evidence for an effect on both wart clearance and reduction in wart recurrence, but in this randomised trial the CIs were wide and the differences did not reach statistical significance.^25^

The HPV DNA prevalence data included in this paper have been published previously.^11^ There are several published reports on the prevalence of HPV infection among MSM in different countries. Two systematic reviews, one considering anal infection and one oral infection, found an association between HPV infection in MSM and HIV seropositivity.^26 27^

It is plausible that MSM not attending a sexual health clinic could have a lower risk of HPV infection, or conversely MSM with limited access to healthcare may have a higher risk of infection and related diseases. The heterogeneity of HPV DNA prevalence and seroprevalence in different settings and countries highlights the importance of determining this in MSM attending sexual health clinics where vaccination may be offered. MSM included in our study were attending a large London-based sexual health clinic and reported high levels of sexual risk behaviour; around two-thirds of men had had more than 30 lifetime male sex partners and over 80% of men have more than 20 male sex partners. MSM in this study may therefore be at higher risk of HPV infection than MSM attending some other clinics, or those not attending other health services, such as primary care. The proportion of MSM with no evidence of a current or previous HPV infection may therefore be a minimum estimate of those likely to benefit from vaccination. However, one limitation of this study was the relatively low number of MSM who were known to be HIV-positive, who have a higher prevalence of HPV DNA and HPV seropositivity. Another limitation was that we did not have sufficient power to compare seropositivity rates with prevalent HPV infection at specific anatomical sites.

The data on the prevalence of HPV DNA infection have informed the mathematical models used to estimate the impact and cost-effectiveness of HPV vaccination of MSM in England.^13^ These models, along with the data on the relationship between seropositivity and DNA detection, may be of interest to other countries considering implementation of a targeted MSM vaccination programme. In the UK, it has subsequently been decided to extend HPV vaccination to adolescent men as part of the National HPV Immunisation Programme. Routine vaccination will be offered to boys aged 12–13 years in a school-based programme, as for the girls. Over time this will start to impact on HPV infection rates in MSM, but this will take decades to substantially benefit the majority of MSM. There is still expected to be continued benefit in having a targeted MSM programme to reduce rates in older MSM and those who missed getting vaccinated in school or who have moved to the UK after age 13.

These data provide information on the proportion of men attending sexual health clinics who are likely to benefit from vaccination. It is encouraging that, in our study, the majority of MSM aged 18–40 years old have no detectable HPV DNA and no serological evidence of exposure to HPV infection despite reporting high rates of sexual risk behaviours. This supports the rationale for opportunistic HPV vaccination of MSM attending sexual health clinics.

**Key messages**

- From April 2018, human papillomavirus (HPV) vaccination has been offered to men who have sex with men (MSM) aged up to 45 years old attending sexual health clinics and HIV clinics in England.
- We present the results from a cross-sectional study including 484 MSM aged 18–40 years old attending a sexual health clinic in London between 2010 and 2012.
- HPV16 and HPV18 DNA prevalence was 13.2% and 6.2%, respectively.
- Seropositivity for HPV16 and HPV18 was 28.5% and 17.1%, respectively; 11.4% seropositive for both types.
- Over half of the MSM in this study had no evidence of a previous or current infection with either HPV16 or HPV18.

## Data Availability

Data are available upon reasonable request.
